# The Relationship between Water Temperature and the Development Cycle Beginning and Duration in Three Black Fly Species

**DOI:** 10.1673/031.013.0101

**Published:** 2013-01-08

**Authors:** Rasa Bernotiene, Galina Bartkeviciene

**Affiliations:** 1 Nature Research Centre, Akademijos 2, LT-08412 Vilnius - 21, Lithuania

**Keywords:** hatching, larvae, pupae, Simuliidae, temperature

## Abstract

Understanding environmental factors affecting the timing and rate of animal development, as well as the factors that cause their effects, is of great importance. The purpose of this study was to establish the relationship between the onset and duration of the development from egg to pupal stage and water temperature in three black fly (Diptera: Simuliidae) species: *Simulium (Simulium) reptans* (Linnaeus 1758), *Simulium (Byssodon) maculatum* (Meigen 1804), *Simulium (Boophthora) erythrocephalum* (De Geer 1776). The study was based on surveys conducted between April and June of 1998–2010. The water temperature on the day of larval eclosion had no statistically significant impact on the beginning of development in any of the three species studied. The date when water temperature in the river reaches a certain value is important to the initiation of development in some black fly species. The present study revealed that the most important dates to the beginning of development of *S. reptans* black flies are when water temperature rises above 5° C, 7° C, and 10° C, while pivotal dates to the development of *S. maculatum* are when water temperature exceeds 4° C and 10° C. Water temperature most often exceeds the value important to the start of the development of these black fly species during March and April. The findings of the present study show that the hatching time of the two black fly species is also related to the mean water temperature in March and April. There were no statistically significant relations established between certain temperature dates and the beginning of larval development in *S. erythrocephalum.* Significant relations (*p* < 0.01) were found to exist between the duration of the development cycle from the first instar larva to pupa and the mean water temperature during the development period in *S. reptans* (r = -0.84; y = 53.088e^-0.0806x^, R^2^ = 0.70), *S. maculatum* (r = -0.82; y = 186.48e^-0.1123x^, R^2^ = 0.69) and *S. erythrocephalum* (r = -0.83; y = 58.768e^-0.0652x^, R^2^ = 0.70). The present study showed that the duration of development from the first instar larva to pupa in all the three black fly species studied was shorter when water temperatures during the development period were higher and longer when water temperatures were lower. The devised model of dependence between the duration of the studied black fly species' development and water temperature was verified experimentally.

## Introduction

It is important to develop an understanding of how living organisms initiate and regulate their development relative to the environment. Water temperature is an ecological factor determining conditions for the existence of hydrobionts (Odum 1986; Caissie 2006).

The objects of this investigation were black flies (Diptera: Simuliidae), which are small, bloodsucking, dipteran insects. Understanding black fly development is important because their bites are painful and they can be present in enormous numbers. More importantly, they are vectors of a number of pathogens that cause onchocercosis, anaplasmosis, tularaemia, myxomatosis, and other diseases (Budaeva and Khitsova 2010). They have a life cycle with an aquatic larval stage, and their larvae are found attached to stones or vegetation in running water. The knowledge of factors influencing black fly development rates will help predict the development of these insects and facilitate the effective use of biotechnical control measures designed for the reduction of black fly abundance.

Temperature is an important factor affecting larval eclosion, development, and emergence. The duration of development from egg to adult in Simuliidae varies with species and depends on water temperature (Crosskey 1990). The purpose of this study was to investigate the relationship between the development onset and duration, and water temperature in three black fly species, namely *Simulium (Simulium) reptans* (Linnaeus) (Diptera: Simuliidae), *Simulium (Byssodon) maculatum* (Meigen) , and *Simulium (Boophthora) erythrocephalum* (De Geer), which start developing in large rivers in Lithuania in the spring.

## Materials and Methods

### Study area

Lithuania lies in the northern part of a temperate climate zone characterized by cold winters and mild summers. The mean annual duration of sunshine in the investigated territory is 1700–1750 hr, the mean annual air temperature is 6.1° C (-5.8° C in January and 16.9° C in July). Snowfall is common in winter months (Galvonaite et al. 2007). Larvae and pupae of black flies were collected in the Nemunas River, in Druskininkai (54° 1′ 10″ N, 23° 58′ 20″ E). The Nemunas River, which is 937 km long, is the largest river in Lithuania and the 14^th^ largest river in Europe. The average discharge of the river near Druskininkai is 213 m^3^/s, its depth is up to 5 m, and stream velocity is 1–2 m/s (Gailiušis et al. 2001).

### Study object

The development of *S. reptans, S. maculatum,* and *S. erythrocephalum* was investigated, which develop abundantly in the Nemunas River in spring (Bernotienė 2003; Bartninkaitė et al. 2006). *S. reptans* and *S. maculatum* black flies have one to two generations per year (Žygutienė and Sprangauskaitė 1998) and overwinter as eggs in both Lithuania and adjacent countries (Kaplych and Skulovetz 2000). The time of eclosion and the development duration of the first generation of these two species were investigated, as the second generation (July–August) is not numerous or is even not found at all (Žygutienė and Sprangauskaitė 1998). *S. erythrocephalum* has 4–5 generations per year and overwinters as larva. The time of eclosion and the development duration of the second generation of this species were investigated. This generation develops in Lithuania in late spring (Bernotienė 2003). The eclosion of first generation *S. erythrocephalum* was recorded in autumn. First generation pupae, which can be found in March–April, are scarce, and sometimes only solitary specimens can be found.

### Sampling

Samples were collected every 5–7 days from the beginning of April until the end of June from 1998 through 2010. The first larvae of *S. maculatum* and *S. reptans* were usually recorded as occurring in the middle of April. The last pupae of the second generation of *S. erythrocephalum* are usually found until the middle of June (Bernotienė 2003). Uniform, band-shaped leaves of *Glyceria maxima* (Hartm.) were collected from the stream at a depth up to 1.5 m, and the abundance of black fly larvae and pupae per 1 dm^2^ of the leaf surface was determined. Three samples were collected each time, and the mean density of black fly larvae and pupae of each species and each larval instar was determined. As *S. maculatum* and *S. reptans* larvae differ from each other in the postgenal cleft shape, which is evident in first or second instars, and *S. erythrocephalum* larvae have a distinctive pattern of the head capsule (Rubtsov 1956), it was not difficult to identify species of the studied specimens even at their early larval stages. Larval instars were determined using postgenal length measurements (Ross and Merritt 1978; Ross and Craig 1979), and larvae with an egg burster on the cephalic apotome were identified as first instars. The emergence time, larval stage duration (the period between hatching from egg and transformation into pupa), and time of pupal emergence were determined.

### Statistical evaluation

Values of daily water temperatures (Tw, °C) in the Nemunas River at Druskininkai for January–June 1998–2010 were obtained from the Lithuanian Hydrometeorological Service. On the basis of mean daily water temperatures, the dates were established when water temperature exceeds 1° C to 10° C (Tw 1 date to Tw 10 date respectively) and never drops below that limit or its drop is shorter than its preceding rise. In some years, water temperature exceeded Tw 11 date values later than the recorded beginning of black fly species development, so the later dates were not analyzed. Mean monthly (Jan–Apr) and ten-day period (I, II, III) water temperatures were analyzed in order to determine the relationship between temperature and the onset of black fly development.

To analyze larval stage duration (the period between hatching from the egg and transformation into pupa), the mean water temperature was computed for that period. Dates of Tw, larval eclosion, and pupal formation of *S. reptans, S. maculatum*, and *S. erythrocephalum* were recalculated in terms of the number of days from January 1. Upper and Lower Quartiles were computed for each parameter investigated. If the appearance of black fly larvae in a particular year exceeded the Upper Quartile limit, it was considered to be late; if it was below the Lower Quartile limit, their appearance in that year was considered to be early.

Spearman's correlation analysis (r = correlation coefficient) was performed to determine statistically reliable relationships between the aforementioned parameters. Multiple regression (a = intercept, b = regression coefficient) analysis was carried out to determine the most important relations between dependent and some independent variables. The determination coefficient (R^2^), F-test (*F*) and *p*-level were calculated in order to estimate the statistical reliability of regression. All statistical calculations were made using the STATISTICA 6 package (Statsoft) for Microsoft Windows 2000.

### Tests in the laboratory

On May 14, 2011 (Tw = 11.0° C), *S. reptans* larvae from the Neris River (in Vilnius city, Lithuania) were sampled. Selected first and second instar larvae were placed into two aquaria (100 specimens in each). In one aquarium water temperature was maintained at 10–11° C, and in the other aquariam it was maintained at 25–26° C. The duration of development from first and second instar larvae to pupal stage was checked in both aquaria.

## Results

### Beginning of development

**Figure 1.**
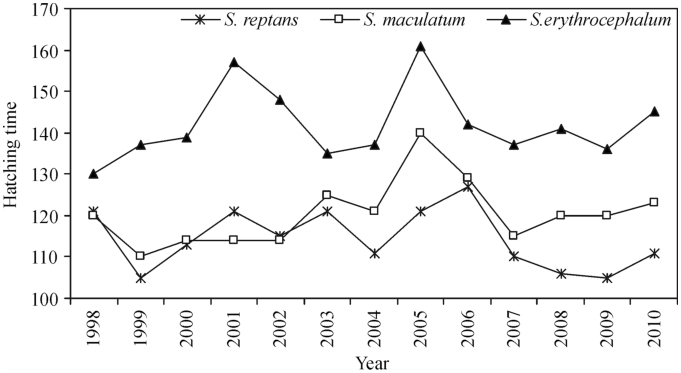
Hatching time (number of days from January 1 to the first day of hatching) of *Simulium reptans, Simulium maculatum,* and *Simulium erythrocephalum* each year from 1998 to 2010. High quality figures are available online.

On the basis of 1998–2010 data, it was established that first instar *S. reptans* were found on day 114.4 ± 7.2 (SE) on average, i.e., April 23 and 24. First instars of *S. maculatum* were found on day 120.4 ± 7.9, i.e., April 29 and 30, while *S. erythrocephalum* larvae were found on day 141.9 ± 8.9, i.e., May 20 and 21. The onset of larval development in all the three species considerably fluctuated in different years: from day 105 to 127 (*S.reptans*), from day 110 to 140 (*S. maculatum*), and from day 130 to 161 (*S. erythrocephalum*) (Figure 1).

Water temperature on the day of larval emergence was on average 12.9 ± 0.6° C (*S. reptans*), 13..7 ± 0.5° C (*S. maculatum*), and 16.3 ± 0.7° C (*S. erythrocephalum*). This temperature sharply fluctuated in different years: from 10.0 to 16.4° C on the day of *S. reptans* larvae emergence, from 9.9 to 17.0° C on the day *S. maculatum* larvae emerged, and from 10.6 to 20.4° C on the day of *S. erythrocephalum* larval emergence. It should be noted that water temperature on the day of larval emergence had no significant impact on the onset of development of any of species studied.

### Dates when water temperature exceeds a certain value

**Figure 2.**
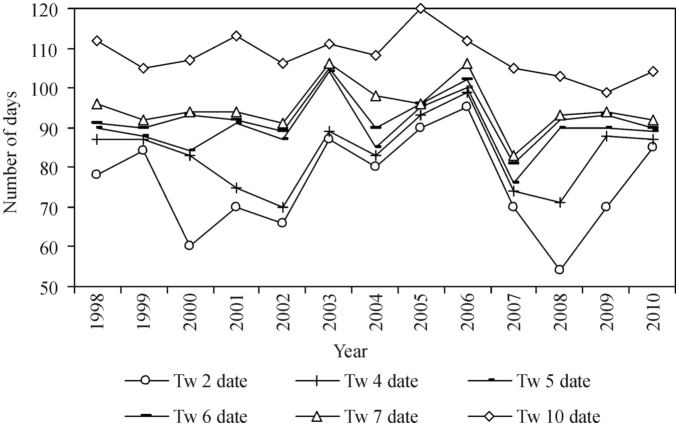
Dates (number of days from January 1) of water temperatures (Tw) in the Nemunas River (Druskininkai) exceeding a certain temperature limit from 1998 through 2010. High quality figures are available online.

The data showed that the date when water temperature exceeded a certain value (Figure 2) was important to the beginning of some black fly species' development. Figure 2 shows the Tw dates that are significantly related to the onset of black fly development. A statistically significant correlation (*p* < 0.05) was found to exist between the beginning of *S. reptans* larval development and dates Tw 5 (r = 0.60), Tw 6 (r = 0.58), Tw 7 (r = 0.62), and Tw 10 (r = 0.83). The present study also revealed a statistically significant relationship (*p* < 0.05) between the onset of *S. maculatum* development and the dates when water temperature exceeded 2° C (r = 0.59), 4° C (r = 0.62), 5° C (r = 0.59), and 10° C (r = 0.61). No statistically significant relations were found to exist between Tw dates and the start of development of *S. erythrocephalum* larvae, although the positive correlation coefficient between the development start of this species larvae and Tw 10 date (r = 0.52) was close to the significance limit.

**Table 1.**
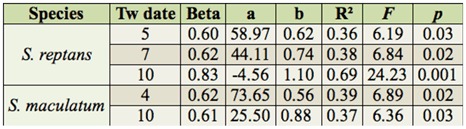
Multiple regression of Tw date on the beginning of *Simulium reptans, Simulium maculatum* larval development.

The multiple regression analysis showed that the most important dates to the initiation of *S. reptans* development were those when water temperature rose above 5° C, 7° C, and 10° C, while the most important dates to the development of *S. maculatum* were those when the water temperature exceeded 4° C and 10° C (Table 1).

The early emergence of *S. reptans* larvae was coincident with early Tw 5 and 10 dates (2008, 2009), Tw 7 and Tw 10 dates (1999). On the contrary, the late emergence of *S. reptans* larvae was coincident with late Tw 5, Tw 7, and Tw 10 dates (2005 and 2006). Similarly, the late appearance of *S. reptans* was coincident with late Tw 7 and 10 dates (1998), Tw 5 and 10 dates (2001), as well as Tw 5 and 7 dates (2003) (Figure 1, 2).

The early appearance of *S. maculatum* larvae from 1999–2002 was coincident with early Tw 2 and Tw 4 dates (2000, 2002). In 2003, 2005, and 2006, the emergence of *S. maculatum* larvae was late, and so were Tw 2, Tw 4, and Tw 10 dates. In regards to *S. erythrocephalum,* it is difficult to trace a connection between its emergence and Tw dates. In 2005, *S. erythrocephalum* emerged late, which coincided with late spring and late Tw dates. However, in 2003, despite the fact that all Tw dates were late, the emergence of *S. erythrocephalum* larvae occurred early (Figure 1, 2).

### Monthly water temperatures

**Figure 3.**
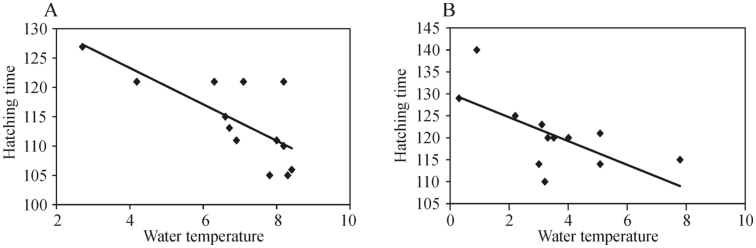
Dependence of the hatching time (days) of *Simulium reptans* (A) on water temperature in the Nemunas River (Druskininkai) in the first ten-day period of April, and that of *S. maculatum* (B) on water temperature (° C) in the third ten-day period of March 1998–2010. High quality figures are available online.

The multiple regression analysis of the relationship between the onset of black fly development and water temperatures in separate months (January–April) as well as in ten-day periods (I, II, III) showed that the beginning of black fly development was dependent on the water temperature in March and April (Table 2, Figure 3). A statistically significant relationship was found to exist between the onset of *S. reptans* development and the mean water temperature in the third ten-day period of March, the first and the second ten-day period of April, and the mean temperature of the whole month of April (Table 2). The closest relationship was established between the beginning of black fly development and water temperature in the first ten-day period of April (Figure 3 A, R^2^ = 0.55). It is at this time that water temperature most often (in 10 years out of 13) exceeded 7° C. A statistically significant relationship was found to exist between the beginning of *S. maculatum* development and the Tw of the third ten-day period of March, the third ten-day period of April, and the mean water temperature of the whole month of March (Table 2). The closest relationship was established between the onset of black fly development and the Tw of the third ten-day period of March (Figure 3B, R^2^ = 0.45), when water temperature most often (8 years out of 13) exceeded the limit of 4° C. At higher water temperatures, the development of *S. reptans* and *S. maculatum* black flies started earlier, and at lower temperatures, the development started later.

**Table 2.**
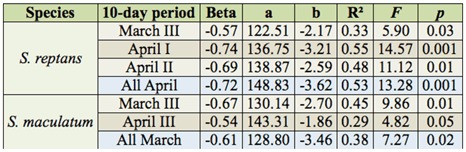
Multiple regression of water temperature in tenday periods (I, II, III) of March and April on the beginning of *Simulium reptans* and *Simulium maculatum* larval development.

### Daily water temperatures

**Figure 4.**
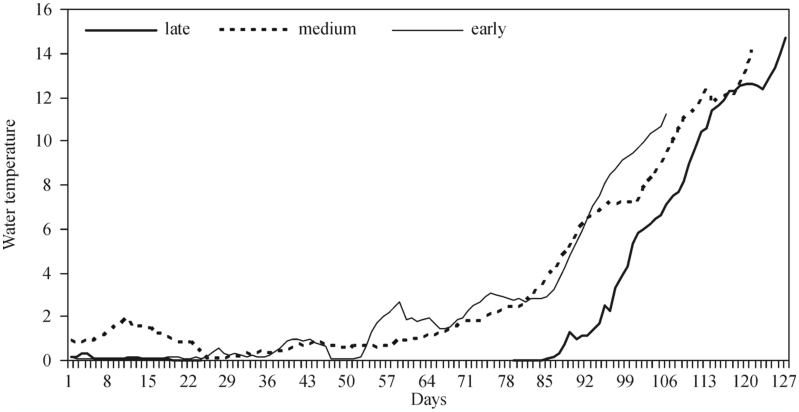
Water temperature (° C) in the Nemunas River (Druskininkai) in the years when the emergence of *Simulium reptans* larvae was late (2006), medium, and early (1999, 2008, 2009). High quality figures are available online.

Results obtained from the analysis of the daily Tw course when black fly development was early (< Lower Quartile), medium, and late (> Upper Quartile) confirm the correlation between high water temperatures and early development, and vice versa. In 2006, *S. reptans* appeared late. Spring arrival was also late, and it was only on the 86^th^ day that water temperature started rising above zero. Water temperature reached 1° C 35 days later compared to the year when the emergence of black flies was early (1999, 2008, 2009) and 26 days later compared to the years when the emergence was medium. Later these differences disappeared, but they persisted until the emergence of *S. reptans* larvae (Figure 4).

**Figure 5.**
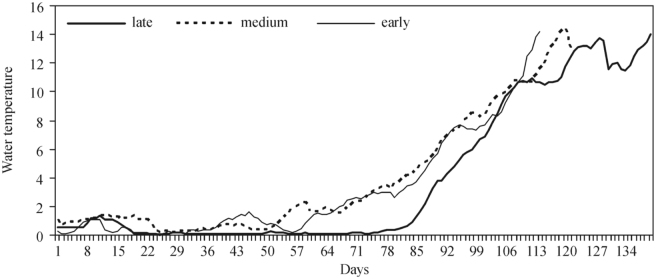
Water temperature (° C) in the Nemunas River (Druskininkai) in the years when the emergence of *Simulium maculatum* larvae was late (2001, 2002, 2005), medium, and early (1998, 2003, 2004, 2007, 2009). High quality figures are available online.

**Figure 6.**
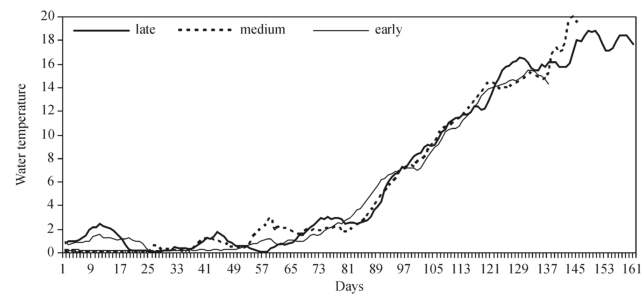
Water temperature (° C) in the Nemunas River (Druskininkai) in the years when the emergence of *Simulium erythrocephalum* larvae was late (2003, 2005, 2006, 2010), medium, and early (1999, 2000, 2001, 2002). High quality figures are available online.

In the years when the beginning of *S. maculatum* larvae development was late (2003, 2005, 2006, 2010), Tw reached 1° C 25 days later and exceeded 2° C 20 days later compared to the years of the early development of black flies (1999, 2000, 2001). In the above-mentioned years, Tw exceeded 4 ^°^C only 6 days later. The analysis of the Tw course revealed that the abovementioned differences persisted until water temperature reached 7° C (8 days) and disappeared later (Figure 5). No significant differences between the early and medium development of *S. reptans* and *S. maculatum* larvae were recorded in the course of Tw (Figure 4, 5). The analysis of the daily course of Tw in relation to the early, close to the medium, or late appearance of *S. erythrocephalum* revealed no difference, and temperature curves were found to overlap (Figure 6).

### Development duration

**Figure 7.**
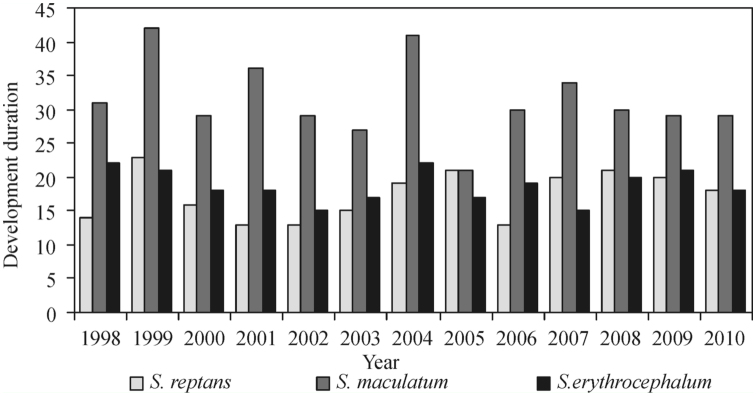
Larval stage duration (days) of *Simulium reptans, Simulium maculatum,* and *Simulium erythrocephalum* black flies in 1998– 2010. High quality figures are available online.

**Figure 8.**
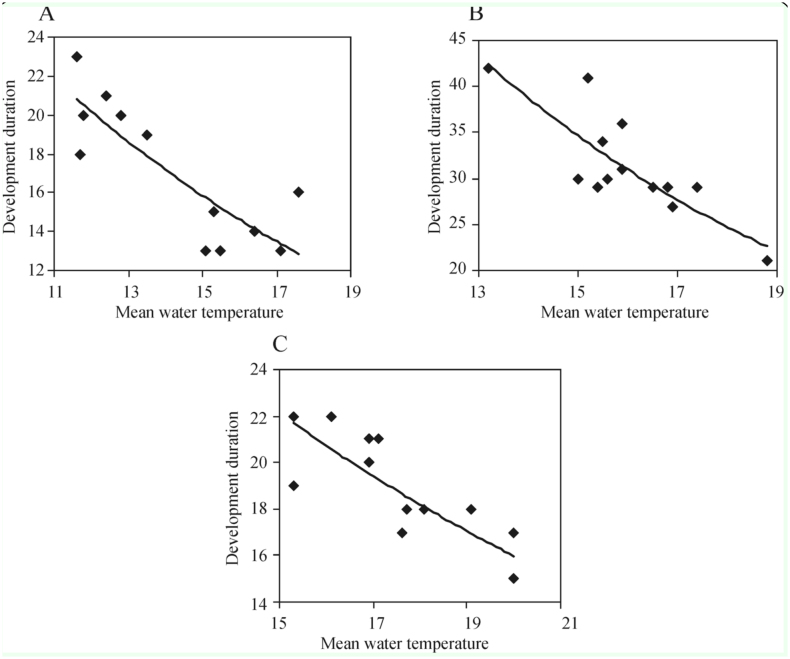
Dependence of the larval stage duration (days) of *Simulium reptans* (A), *Simulium maculatum* (B), and *Simulium erythrocephalum* (C) on the mean water temperature (° C) during the development period in the Nemunas River (Druskininkai) in 1998–2010. High quality figures are available online.

From 1998–2010, the emergence of *S. reptans* pupae was recorded on day 131.8 ± 1.4 (SE) on average, i.e., on May 11 and 12, that of *S. maculatum* pupae on day 151.8 ± 1.6, i.e., on May 31 or June 1, and that of *S. erythrocephalum* pupae on day 160.6 ± 2.2, i.e., on June 9 and 10. The interval between the first appearance of larvae and the first appearance of pupae each year varied from 13 (2001, 2002, 2006) to 23 days (mean 17.4 ± 1.0) for *S. reptans,* from 21 (2005) to 42 days (1999) (mean 31.4 ± 1.6) for *S. maculatum,* and from 15 (2002, 2007) to 22 days (1998, 2004) (mean 18.7 ± 0.7) for *S. erythrocephalum* (Figure 7). A significant relationship (*p* < 0.01) was established between the development duration from the first instar larva to pupa and the mean water temperature during the development period in *S. reptans* (r = -0.84; y = 53.088e^-0.0806x^; R^2^ = 0.70), *S. maculatum* (r = -0.82; y = 186.48e^0.1123x^. R^2^ =0.69) and *S.erythrocephalum* (r = -0.83; y = 58.768e^-0.0652x^, R^2^ = 0.70) (Figure 8).

According to the data obtained in the present study, the emergence of *S. reptans* larvae was early in 2008 and 2009 (Figure 1). The development duration in the 2008 and 2009 lasted from 21 to 20 days respectively (Figure 7), the mean Tw was 12.4°C and 12.8° C respectively, and the emergence of pupae occurred early. In 1999, the appearance of the first *S. reptans* larvae was recorded early (Figure 1), but due to the low mean water temperature (11.6° C) the duration of their development into the pupal stage was long (Figure 7). Therefore, the timing of pupal emergence was close to the average. In 2006, the late appearance of larvae was followed by the late appearance of pupae (mean Tw = 15.5° C), the development period being short. The duration of *S. reptans* development to pupa was found to be short in 2001 and 2002 (Figure 7), when the mean water temperature equalled 17.1° C and 15.1° C respectively.

The emergence of *S. maculatum* larvae was recorded early in 2000 and 2002 (Figure 1), and the larval stage duration lasting 29 days was close to the mean (Figure 7). The mean water temperature recorded in those years was 17.4° C and 16.8° C respectively, and the appearance of pupae was early. In 2005 and 2006, the emergence of both *S. maculatum* larvae and pupae was delayed. The duration of the larval stage in 2005 was short (21 days, mean Tw = 18.8° C), while in 2006 it was close to the mean (30 days, mean Tw = 15.6° C). In 2004, the appearance of *S. maculatum* was close to the average, but as pupae emerged late, the development duration was prolonged (41 days, mean Tw = 15.2° C). In 1999, when the mean Tw was the lowest (13.2° C), the larval stage of *S. maculatum* was the longest (42 days). The early appearance of *S. erythrocephalum* larvae (Figure 1) and pupae was recorded in 1998 and 2003, and the period of development to pupa was prolonged, lasting 22 (mean Tw = 16.1° C) and 17 days (mean Tw = 17.6° C) respectively. In 2001 and 2005, *S. erythrocephalum* larvae and pupae appeared late, their development lasting 18 (mean Tw = 18.1° C) and 17 (mean Tw = 20.0° C) days (Figure 7) respectively. In 2002, *S. erythrocephalum* larvae emerged late, but as the development period was short (probably due to the high mean water temperature during the development period (13 days, mean Tw = 20.0° C)), the emergence of pupae was close to the average. The situation in 2007 was similar to 2002, as the emergence of *S. erythrocephalum* pupae was recorded early, the development period was short (15 days), and the mean water temperature was high (20.0° C). In 2009, *S. erythrocephalum* larvae appeared early (Figure 1), but the emergence of pupae was recorded at the usual time because of the protracted development lasting 21 days (mean Tw= 19.1° C).

### Tests in the laboratory

The model of black fly development duration dependence on water temperature used in the present study was tested experimentally. Selected first and second instar *S. reptans* larvae that had been sampled from the river were placed into two aquaria with different water temperatures. In the aquarium with water temperature ranging from 10 to 11° C, the development of pupae lasted for 25 days (according to the model (Figure 8A) the development ought to have lasted 22–24 days). Meanwhile, in the aquarium where water temperature was maintained at 25 to 26° C, not a single *S. reptans* larva survived into the pupal stage. To sum up, the duration of development from larva to pupa in all the three black fly species was shorter when the mean water temperature during this period was higher, and, vice versa, it is prolonged at a lower water temperature (Figures 8A, B, C).

## Discussion

The three black fly species studied, & *reptans, S. maculatum,* and *S. erythrocephalum,* develop abundantly in the Nemunas River in the spring (Bernotienė 2003; Bartninkaitė et al. 2006). These three species differ in their biology. *S. reptans* and *S. maculatum* overwinter as eggs (Kaplych and Skulovetz 2000), while *S. erythrocephalum* overwinters as larvae (Post 1982). *S. erythrocephalum* pupae can be found in small numbers in March–April. The eclosion of the second generation of *S. erythrocephalum* occures in May–June (Bernotienė 2003). According to some data, in Western Europe, the pupal development of the first generation of *S. erythrocephalum* occurs in February (Post 1983). The present study established that larvae of other species, *Simulium posticatum* Meigen 1838, *S. ornatum* Meigen 1818, *S. lineatum* (Meigen 1904), *S. vernum* Macquart 1826, and *S. equinum* (Linnaeus 1758), can be found in spring in the Nemunas River, but they are not abundant and, what is more, they are not found in the river every year.

Differences in the eclosion time of certain black fly species were quite considerable and in some years they reached 22 (*S. reptans*), 30 (*S. maculatum*), and 31 (*S. erythrocephalum*) days. Therefore, it was interesting to elucidate the factors impacting eclosion time fluctuations in different years for each black fly species. Undoubtedly, water temperature exerts the greatest effect on the timing of larval emergence (Rubtsov, 1956; Adler et al. 2004). The findings of the present study are similar to those of other researchers in terms of life cycle duration of black fly species from both Palaearctic and Nearctic regions (Carlsson 1967; Post 1982; McCreadie and Colbo 1991). The present study was conducted on three black fly species, because it was well-known that the dependence of development parameters of each black fly species on temperature was different. For example, the *S. verecundum* species complex begins to suffer severe mortality at temperatures below 10° C (McCreadie and Colbo 1991), whereas the development of *Prosimulium approximatum* is not curtailed until temperature drops below 4° C (Mansingh and Steele 1973; Adler et al. 2004). According to Carlsson (1967), after hibernation, large numbers of eggs hatch as soon as the temperature rises above certain values, which in many species is when water temperature reaches about 8° C. Thus, a springtime maximum in the black fly population occurs, during which the part of the watercourse inhabited by black flies is overcrowded (Carlsson 1967). The analysis of the present study revealed the following temperature ranges on the day larvae of the three black fly species emerged: 10.0–16.4° C for *S. reptans*, 9.9–17.0°C for *S. maculatum*, and 10.6–20.4° C for *S. erythrocephalum.* In spite of the fact that water temperature on the day of larval eclosion from eggs of *S. reptans* and *S. maculatum* specimens was similar, it was only in some years (1998, 2000, 2002, 2006) that these two species eclosed at a similar time. The appearance of & *reptans* larvae in rivers most often (8 years out of 13) preceded that of *S. maculatum*, with the exception of the year 2001, when *S. maculatum* larvae were the first to be detected in rivers. The results of this study revealed that water temperature on the day of larval eclosion is not the main factor affecting hatching time.

Earlier investigations showed that the hatching time of *S. maculatum* eggs in Lithuania was influenced by winter air temperatures, especially those in March (Bernotiene and Bartkeviciene 2011). The mean water temperature in the Nemunas River is usually lower by 1–2° C than the mean air temperature in that month (Kilkus 1998). These results suggest that the onset of black fly development is predetermined by water temperature long before the hatching process starts. Thus, the dates when water temperature rises to 5° C, 7° C, and 10°C are important to the beginning of *S. reptans* development. As far as the onset of *S. maculatum* development is concerned, the pivotal dates are when water temperature reaches 4° C and 10° C (Table 1). These differences in the biology of the two black fly species most likely account for the fact that in some years *S. reptans* larvae appear in the Nemunas River earlier than those of *S. maculatum*, while in other years it is *S. maculatum* larvae that are detected in the river first.

There was no statistically significant relationship established between Tw dates and the beginning of the larval stage in *S. erythrocephalum.* The reason behind this lack of relationship could be the biology of *S. erythrocephalum,* which differs from that of *S. reptans* and *S. maculatum. S. erythrocephalum* black flies overwinter as larva, i.e., they hatch from eggs in autumn, most often in October, and pupate early in spring (Post 1982). The hatching process of the second generation must have been predetermined by temperature of the whole winter season (Odintsov 1961), which influenced larval hibernation and the timing of the formation of the first generation pupae (according to the data from the present study, it varied considerably in different years), the latter impacting the hatching time of the second generation. Investigations of the relationship between the development parameters of *S. erythrocephalum* black flies and water temperature will be continued, but it is evident from the first data that pupae of the first generation are detected at water temperatures in the Nemunas River ranging from 2.5° C (1998) to 10.0° C (2000). These data are not going to be discussed in this paper, as they need separate analysis. However, they extend the knowledge of the biology of *S. erythrocephalum* development. It has been shown (Ruhm 1970) that for the *S. erythrocephalum* spring generation, pupation does not occur below 5° C, mass emergence occurrs when river temperature rose to about 8° C, and pupation is completed below 13°C.

The water temperature at which the embryonic development of an egg begins is not the only factor affecting the time of the first larvae appearance. It is likely that mean water temperature during the period of embryonic development is also a factor. According to Fredeen (1959), eggs of some species hatch in about 4 days at 25° C, but some eggs remain viable for 2.5 years when stored at temperatures slightly above freezing. Ruhm (1969) found the mean incubation period to last eight days at 15.0° C, and Wenk (1966) recorded a 9.7-day incubation period at 12.5° C for *S. erythrocephalum*. Factors such as oxygen tension and photoperiod can also affect the rate of egg development and initiate hatching (Adler et al. 2004).

Larval stage duration can be influenced by river discharge, seston quantity, but it is temperature that exerts the greatest impact on it (Merritt et al. 1982; Hauer and Benke 1987). The recorded differences in the interval between the first appearance of larvae and that of pupae each year was 10 days for *S. reptans*, 21 day for *S. maculatum* and 7 days for *S. erythrocephalum.* Significant relations were found to exist between the larval stage duration and the mean water temperature during the development period in *S. reptans, S. maculatum,* and *S. erythrocephalum.* The differences in mean water temperatures during the black fly development period predetermined different durations of black fly development in different years. The findings of the present study support the conclusions of other researchers that the rate of larval growth is faster at higher temperatures (Post 1982; Crosskey 1990). However, each species has a certain optimum temperature at the larval stage, and this temperature varies considerably depending on species from around 4° C for *Prosimulium* spp. to 12° C for most other species (Carlsson 1967). A rise of 4° C in the mean daily temperature during individual rearing resulted in reductions of the minimum development time by 32% in *S. erythrocephalum* and by 14% in *S. lineatum* (Ham and Bianko 1984). The model of dependence of *S. erythrocephalum* development rate on water temperature (within broad temperature limits 10–22° C) used in the present study confirmed the result reported by Ham and Bianko (1984) because a rise of 4° C in water temperature accelerated the development rate of the species by 32.6% ± 2.92 (SE). According to data available in literature, at a water temperature of 13.2° C, *S. maculatum* took 42 days to develop, while only taking 21 days when water temperature was 18.8 °C (Bernotiene and Bartkeviciene 2011). According to the model used in the present study, the development of this species would last 43 days at a water temperature of 13° C and 22 days at of 19° C.

The model of species development dependence on water temperature was tested experimentally. For that purpose, *S. reptans* larvae were cultivated in aquaria. The duration of larval development at a water temperature of 10–11° C coincided with the one predicted by the model. However, larvae cultivated at a water temperature of 25–26° C did not develop at all, and all of them perished. The reduction of water temperature below the optimum eventually arrests development, the lower limit varying with species (Adler et al. 2004). An increase in water temperature above the optimum may also have considerable effects on the development of black flies, temperature limits varying with different black fly species. In April and May, i.e., in the period of *S. reptans* and *S. maculatum* larval development, water temperature in the Nemunas River very rarely exceeded 20° C. In more exact terms, during the study period in April, water temperature did not exceed 20° C (maximum 19.7° C), while in May, water temperature above 20° C was recorded only on 7% of days (maximum 24.4° C). During the period of *S. erythrocephalum* development, water temperature rises above 25°C (maximum 25.6° C) in some years.

Higher water temperatures may not favor the development of some black fly species. Therefore, (> 25° C) the model used in this study does not seem to be reliable in predicting the black fly development duration at high temperatures. The comparison of development rates of the three black fly species showed that the development of *S. maculatum* was the slowest (31 days at 16° C) and that of *S. reptans* was the fastest (15 days at 16° C). This result may be explained by a different distribution of black fly species; *S. maculatum* black flies are adapted to a continental climate with larvae developing in large warm rivers (Yankovsky 2002), and *S. reptans* black flies are very common in North Europe (Rubtsov 1956).

In conclusion, the date when water temperature reaches a certain value is important to the beginning of *S. reptans* and *S. maculatum* development. It is in March and April that water temperature most often exceeds the value important to the initiation of the development of these two black fly species. The findings of this study showed that the hatching time of two black fly species that hibernate as eggs is related to the mean water temperature in March and April. The duration of the larval stage in all the investigated black fly species depended on the mean water temperature during the development period. The duration of development was shorter when water temperatures were higher, and longer when water temperatures were lower.

## Abbreviations

**Tw**,: water temperature
